# Asciminib Maintains Antibody-Dependent Cellular Cytotoxicity against Leukemic Blasts

**DOI:** 10.3390/cancers16071288

**Published:** 2024-03-26

**Authors:** Samuel J. Holzmayer, Joseph Kauer, Jonas Mauermann, Tobias Roider, Melanie Märklin

**Affiliations:** 1 Clinical Collaboration Unit Translational Immunology, German Cancer Consortium (DKTK), Department of Internal Medicine, University Hospital Tübingen, 72076 Tübingen, Germany; samuel.holzmayer@med.uni-tuebingen.de (S.J.H.);; 2 Cluster of Excellence iFIT (EXC 2180), Image-Guided and Functionally Instructed Tumor Therapies, Eberhard Karls University, 72076 Tübingen, Germany; 3 Interfaculty Institute for Cell Biology, Department of Immunology, University of Tübingen, German Cancer Consortium (DKTK) and German Cancer Research Center (DKFZ), Partner Site Tübingen, 72076 Tübingen, Germany; 4 Department of Hematology, Oncology and Rheumatology, University Hospital Heidelberg, 69117 Heidelberg, Germany; tobias.roider@med.uni-heidelberg.de; 5 European Molecular Biology Laboratory (EMBL), 69116 Heidelberg, Germany

**Keywords:** acute lymphoblastic leukemia, bcr-abl, asciminib, monoclonal antibodies, TKI

## Abstract

**Simple Summary:**

Acute lymphoblastic leukemia is a malignant disease which is commonly treated with various chemotherapeutic drugs. Novel therapeutic options are gaining interest, most of them involving therapeutic antibodies. In a high-risk genetic subset called BCR-ABL1 positive acute lymphoblastic leukemia, tyrosine kinase inhibitors are successfully used. Tyrosine kinase inhibitors combine well with chemotherapy but might interfere with antibody therapy. The effects of tyrosine kinase inhibitors on antibody dependent cellular cytotoxicity are not fully understood. We therefore tested a novel inhibitor called asciminib that is tested in acute lymphoblastic leukemia and assessed its influence on immune cell activation by antibodies. We found that asciminib, in contrast to other agents such as dasatinib, does not interfere with antibody therapy and should therefore be tested in clinical trials for patients with acute lymphoblastic leukemia.

**Abstract:**

B cell acute lymphoblastic leukemia (B-ALL) is characterized by an accumulation of malignant precursor cells. Treatment consists of multiagent chemotherapy followed by allogeneic stem cell transplantation in high-risk patients. In addition, patients bearing the BCR-ABL1 fusion gene receive concomitant tyrosine kinase inhibitor (TKI) therapy. On the other hand, monoclonal antibody therapy is increasingly used in both clinical trials and real-world settings. The introduction of rituximab has improved the outcomes in CD20 positive cases. Other monoclonal antibodies, such as tafasitamab (anti-CD19), obinutuzumab (anti-CD20) and epratuzumab (anti-CD22) have been tested in trials (NCT05366218, NCT04920968, NCT00098839). The efficacy of monoclonal antibodies is based, at least in part, on their ability to induce antibody-dependent cellular cytotoxicity (ADCC). Combination treatments, e.g., chemotherapy and TKI, should therefore be screened for potential interference with ADCC. Here, we report on in vitro data using BCR-ABL1 positive and negative B-ALL cell lines treated with rituximab and TKI. NK cell activation, proliferation, degranulation, cytokine release and tumor cell lysis were analyzed. In contrast to ATP site inhibitors such as dasatinib and ponatinib, the novel first-in-class selective allosteric ABL myristoyl pocket (STAMP) inhibitor asciminib did not significantly impact ADCC in our settings. Our results suggest that asciminib should be considered in clinical trials.

## 1. Introduction

B cell acute lymphoblastic leukemia (B-ALL) is a malignancy characterized by the proliferation of transformed precursor B cells. A genetic subgroup indicating high-risk disease is defined by BCR-ABL1-fusion-gene-positive patients [1,2]. Such patients are treated with multi-agent chemotherapy combined with tyrosine kinase inhibitors (TKI) and consolidated with allogeneic stem cell transplantation [3,4,5,6]. The introduction of TKI has improved 5-year overall survival to 70–80% [4,6], compared to 30–40% in the pre-TKI era [7]. However, novel protocols reduce chemotherapy burden by utilizing bispecific or monospecific antibodies thus aiming at an immunotherapeutic effect [8,9,10,11]. One such strategy is the use of monospecific antibodies that elicit antibody-dependent cellular cytotoxicity (ADCC) via their Fc region. Rituximab improves outcomes in CD20-positive cases [12,13]. Different antibodies, such as tafasitamab (anti-CD19) [14], isatuximab (anti-CD38) [15], epratuzumab (anti-CD22) [16] and obinutuzumab (anti-CD20), have been tested in clinical trials (NCT05366218, NCT04920968, NCT00098839). If proven effective in B-ALL, they might also be used in combination with TKI in BCR-ABL1-positive cases. However, TKI used concomitantly may interfere with ADCC [17,18]. Therefore, a combinatorial regimen should be tested for potential interference with the desired immunotherapeutic effects. Here, we focused on currently available TKI for BCR-ABL1-positive ALL, including bosutinib, imatinib, dasatinib, nilotinib, ponatinib and the novel first-in-class selective allosteric ABL myristoyl pocket (STAMP) inhibitor asciminib, which is currently being tested for BCR-ABL1-positive ALL, but not yet approved [19]. We characterize the effect of each TKI on NK cell activity by measuring activation, degranulation, proliferation, cytokine release and tumor cell killing. Our results support the inclusion of asciminib in clinical trials for BCR-ABL1-positive ALL patients due to its non-interference with immunotherapy.

## 2. Materials and Methods

### 2.1. Peripheral Blood Mononuclear Cells and Cell Lines

All experiments were performed according to the Helsinki protocol and approved by the Ethics Committee of the University of Tübingen (vote: 13/2007V). Informed consent was obtained from all enrolled participants. Peripheral blood samples were collected from healthy donors, and peripheral blood mononuclear cells (PBMCs) were isolated using density gradient centrifugation with Biocoll Cell Separation Solution (Biochrom, Berlin, Germany). The B-ALL cell lines Nalm-6, Nalm-16, SD-1 and TOM-1 were obtained from the German Collection of Microorganisms and Cell Cultures (DSMZ, Braunschweig, Germany) and routinely screened for mycoplasma contamination. PBMCs and cell lines were cultured in RPMI 1640 medium, GlutaMAX Supplement (Life Technologies, Darmstadt, Germany) supplemented with 10% heat-inactivated fetal calf serum (PAN-biotech, Aidenbach, Germany), 100 U/mL penicillin (Sigma-Aldrich, St. Louis, MO, USA) and 100 μg/mL streptomycin (Sigma-Aldrich), at 37 °C with 5% CO_2_.

### 2.2. Tyrosine Kinase Inhibitors (TKI)

Asciminib, (ASCI), bosutinib (BOSU), dasatinib (DASA), imatinib (IMA), nilotinib (NILO) and ponatinib (PONA) were obtained from Selleckchem (Cologne, Germany). The TKI were dissolved in DMSO and stored at −80 °C. Stock concentrations were 5 mM for ASCI, BOSU, DASA and PONA, and 10 mM for IMA and NILO. Peak and IC50 concentrations were chosen based on clinical studies which reported serum levels in patient samples. Peak levels as well as steady-state concentrations (from here on: IC50) were based on these data [20,21,22,23,24,25,26,27]. The concentrations were chosen as follows: ASCI 806 nM (peak) and 28 nM (IC50); BOSU 600 nM (peak) and 2.4 nM (IC50); DASA 150 nM (peak) and 10 nM (IC50); IMA 3000 nM (peak) and 600 nM (IC50); NILO 3000 nM (peak) and 30 nM (IC50); PONA 145 nM (peak) and 70 nM (IC50). DMSO was included as a control in all experiments at a maximum concentration of 0.1% *v*/*v* in NILO peak samples.

### 2.3. Flow Cytometry

Antibodies including CD3-FITC (clone OKT3; 1:150, BioLegend, San Diego, CA, USA), CD56 PE/Cy7 (clone NCAM1; 1:50, BioLegend), CD69-PE (clone FN50; 1:60, BD Biosciences, Heidelberg, Germany), CD107a-PE (clone H4A3; 1:25, BD Biosciences) and the corresponding isotype controls were obtained from BioLegend. 7-AAD (BioLegend) staining (1:200) or LIVE/DEAD™ Fixable Violet (1:1000, Thermo Fisher Scientific, Waltham, MA, USA) were used to identify non-viable cells within flow cytometric samples. For flow cytometric assays, 30,000 target cells were incubated in 96-well plates with 65,000 PBMCs, Rituximab at 2.5 µg/mL and various tyrosine kinase inhibitors at the indicated concentrations. After 4 and 24 h, flow cytometric analyses were performed to determine CD107a and CD69 expression, respectively. NK cells were identified as CD56+ CD3+, activated subsets as CD69+. CD107a expression indicated degranulation. Detailed gating strategies are provided in Supplementary Figure S1. All samples were analyzed using the BD FACS Canto II or BD FACS Fortessa II (BD Biosciences, Heidelberg, Germany). Data analysis was performed using FlowJo V10 software (BD Bioscience).

### 2.4. Cytotoxicity Assays

Lysis of cell lines by allogeneic PBMC was analyzed using a 2 h BATDA Europium assay as previously described. Long-term cytotoxicity analyses were performed using the IncuCyte^®^ S3 Live Cell Analysis System (Essenbioscience, Sartorius, Göttingen, Germany). B-ALL cells were labeled with Incucyte^®^ Nuclight Rapid Red Dye (Sartorius, Germany, Göttingen) seeded in 96-well plates and cocultured with PBMC from healthy donors (E:T ratio 20:1) with or without the indicated mAbs (2.5 µg/mL each). To determine the amount of red labeled B-ALL cells, images were taken at 10× magnification every 4 h for 120 h. Quantification of viable cells was performed through normalization to the corresponding measurement at seed. The number of cells at seed was set to 100%.

### 2.5. Legendplex Cytokine Arrays

Legendplex cytokine arrays (BioLegend) for the measurement of granzyme A, perforin, sFasL, TNF, IL 2, IFNγ, IL 6 and IL 10 using Legendplex assays were performed with supernatants from 24 h co-culture experiments of B-ALL cells with PBMC from healthy donors (E:T ratio 5:1) according to the manufacturer’s protocol.

### 2.6. Statistical Analysis

Data are presented as bar graphs with means superimposed by dot plots or box plots with min/max whiskers and dot plots for individual values. ANOVA tests with Bonferroni correction were used for group comparisons. Statistical analyses were performed using Prism 8.1.0 (GraphPad Software, Boston, MA, USA), R 4.3.1 and SPSS 29 (IBM, Armonk, NY, USA) software. *p*-values < 0.05 were considered statistically significant.

## 3. Results

Various in vitro assays were performed to elucidate the effect of different TKI on NK cell activation, degranulation, proliferation and NK-mediated tumor cell lysis.

### 3.1. NK Cell Activation in the Presence of TKI

**Figure 1:**
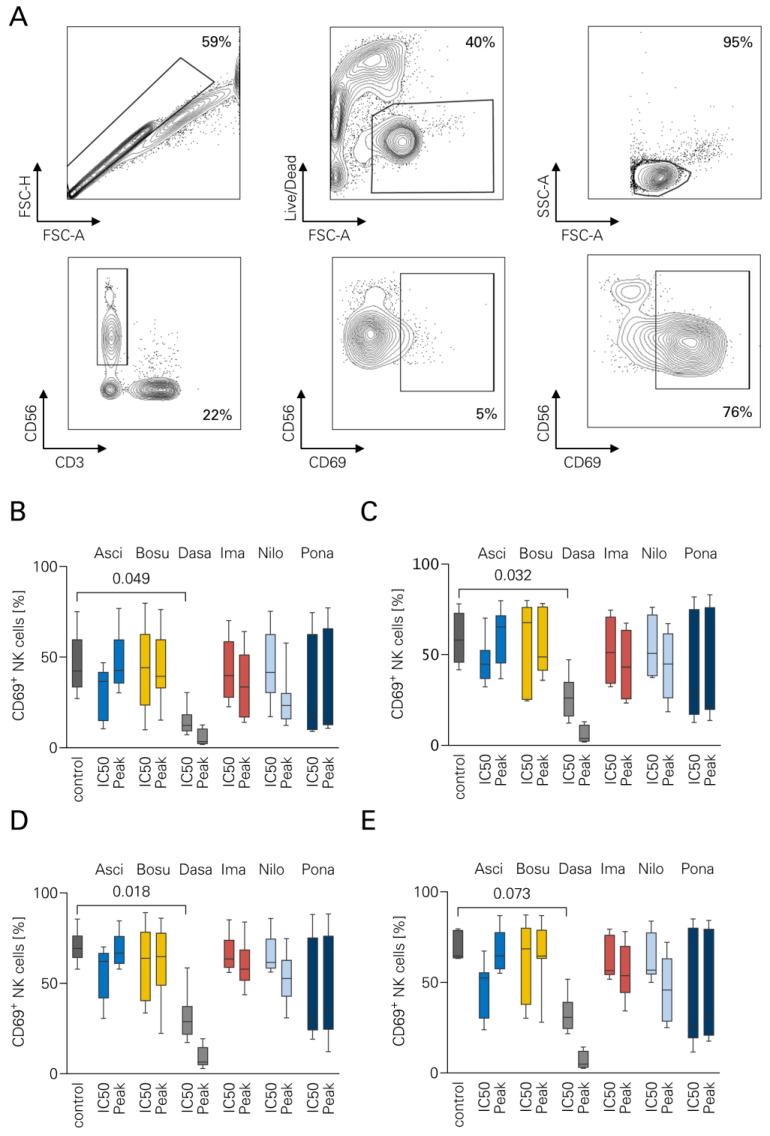
NK cell activation in the presence of TKI. ALL cell lines were cultured for 24 h together with PBMCs (*n* = 7) and Rituximab +/− different TKI (Asciminib, Bosutinib, Dasatinib, Imatinib, Nilotinib, Ponatinib). CD69 expression as activation marker was measured using flow cytometry. (**A**) Exemplary gating strategy: Singlets–live cells–lymphocytes–CD56+CD3- NK cells–CD56+CD69+ activated subsets. The following cell lines were used: (**B**) BCR-ABL1-negative Nalm-6. (**C**) BCR-ABL1 negative Nalm-16. (**D**) BCR-ABL1 positive SD-1. (**E**) BCR-ABL1-positive TOM-1. Boxplots with Tukey whiskers. ANOVA with Bonferroni correction.

To evaluate NK cell activation in the presence of TKI, flow cytometry-based assays were performed with PBMC from healthy donors (*n* = 7) (Figure 1). All experiments were performed using two bcr-abl negative cell lines (Nalm-6, Nalm-16) and two bcr-abl positive cell lines (SD-1, TOM-1). In Nalm-6, DASA resulted in a significantly lower CD69 expression (10.3% vs. 45.7%, *p* = 0.045) (Figure 1B). Similarly, DASA reduced CD69 in Nalm-16 to 16.1% compared to the control (58.4%, *p* = 0.032) (Figure 1C). In SD-1 cells, the effects of DASA were comparable, showing 20.2% activated NK cells vs. 70.3% in control wells (*p* = 0.018) (Figure 1D). A similar trend was observed in TOM-1 cells (19.2% vs. 68.5%), which did not reach significance (*p* = 0.073) (Figure 1E). None of the other TKI induced significant differences compared to the control group.

### 3.2. NK Cell Proliferation and TKI

**Figure 2:**
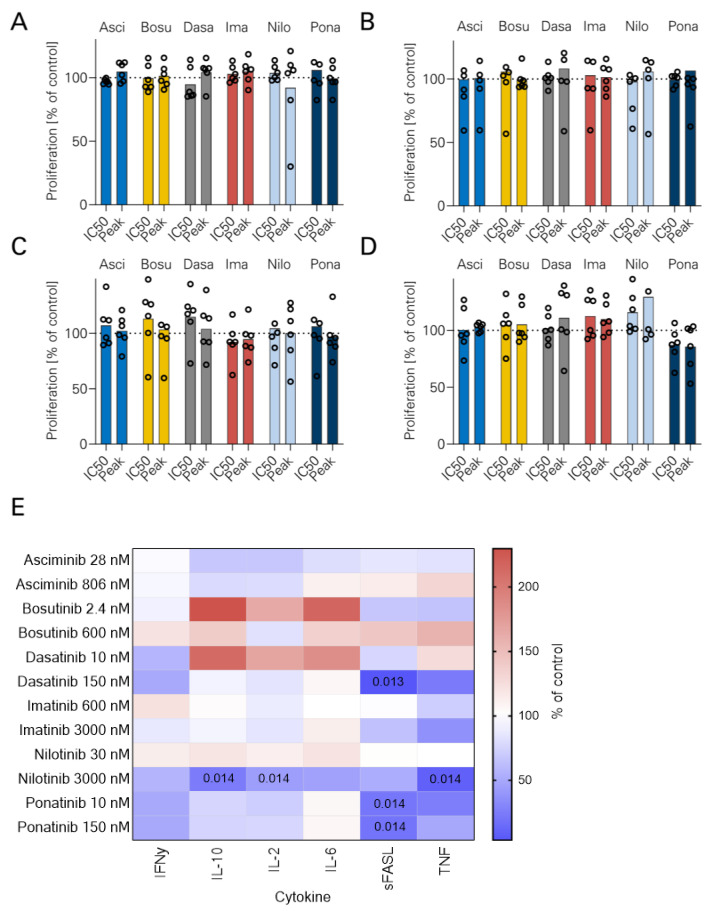
NK cell proliferation and cytokine release in the presence of TKI. ALL cell lines were cultured for 24 h together with PBMCs (*n* = 6) and Rituximab +/− different TKI (Asciminib, Bosutinib, Dasatinib, Imatinib, Nilotinib, Ponatinib). Absolute NK cell counts were measured using flow cytometry. The following cell lines were used: (**A**) BCR-ABL1-negative Nalm-6. (**B**) BCR-ABL1 negative Nalm-16. (**C**) BCR-ABL1 positive SD-1. (**D**) BCR-ABL1-positive TOM-1. Bar graphs (mean) with individual values. (**E**) After 24 h, supernatants from the abovementioned experiments (*n* = 4 PBMC donors) were analyzed using a LEGENDplex assay. IFNy, IL-2, IL-6, IL10, sFASL and TNF levels were measured. Results obtained with the TOM-1 cell line are shown. ANOVA with Bonferroni correction.

Next, NK cell proliferation was measured using flow cytometry after 24 h stimulation with B-ALL cells with rituximab with or without TKI. No significant reduction of absolute NK cell numbers was observed with either TKI (Figure 2A–D). Additionally, cytokine secretion by NK cells was measured using LEGENDplex assays after 24 h stimulation with rituximab (Figure 2E). NILO at peak levels reduced IL-10 (2.0 versus 16.5 pg/mL, *p* = 0.014) and IL-2 secretion (2.0 vs. 16.5 pg/mL, *p* = 0.014) as well as TNF production (10.0 vs. 145.0 pg/mL, *p* = 0.014). PONA inhibited sFASL secretion at both IC50 and peak levels (3.77 and 3.48 vs. 17.5 pg/mL, respectively; *p* = 0.014). Addition of DASA at peak levels significantly decreased sFASL secretion (0.73 vs. 17.5 pg/mL, *p* = 0.013). The latter findings suggest an inhibitory effect of SRC–ABL TKI on sFASL-mediated cell death.

### 3.3. NK Cell Degranulation and TKI

**Figure 3:**
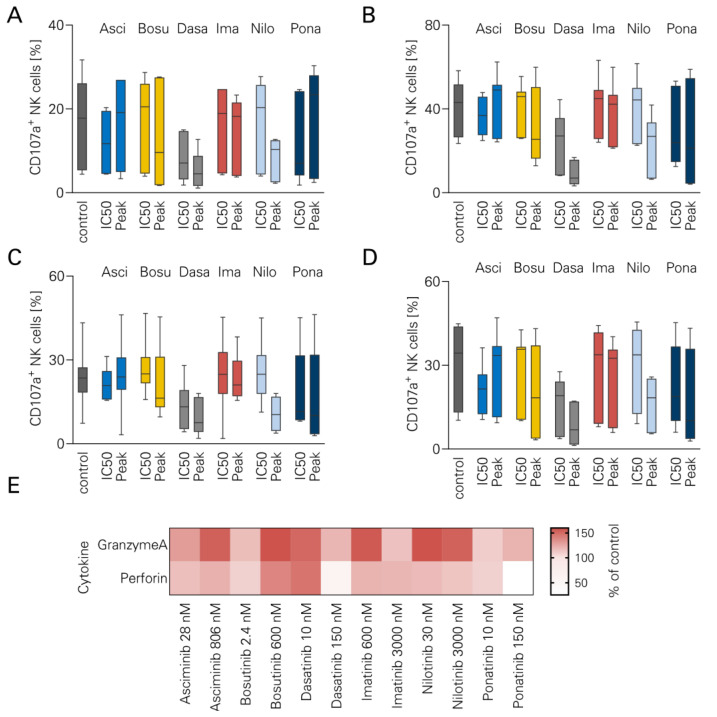
NK cell degranulation in the presence of TKI. ALL cell lines were cultured for 24 h together with PBMCs (*n* = 7) and Rituximab +/− different TKI (Asciminib, Bosutinib, Dasatinib, Imatinib, Nilotinib, Ponatinib). CD107a expression as degranulation marker was measured using flow cytometry. The following cell lines were used: (**A**) BCR-ABL1-negative Nalm-6. (**B**) BCR-ABL1-negative Nalm-16. (**C**) BCR-ABL1 positive SD-1. (**D**) BCR-ABL1-positive TOM-1. Boxplots with Tukey whiskers. (**E**) After 24 h, supernatants from the abovementioned experiments (*n* = 4 PBMC donors) were analyzed using a LEGENDplex assay. Granzyme A and perforin levels were measured.

To evaluate the influence of TKI on NK cell degranulation and effector molecule secretion, CD107a levels were measured using flow cytometry after 24 h stimulation with rituximab. The gating strategy is shown in Supplementary Figure S1. No significant reduction in CD107a expression was observed with either TKI (Figure 3A–D). To validate these findings, LEGENDplex cytokine arrays were performed using supernatants from the abovementioned assays. Consistent with the preserved CD107a expression, secretion of granzyme A and perforin was not affected by either TKI (Figure 3E).

### 3.4. Short-Term Lysis of Tumor Cells

**Figure 4:**
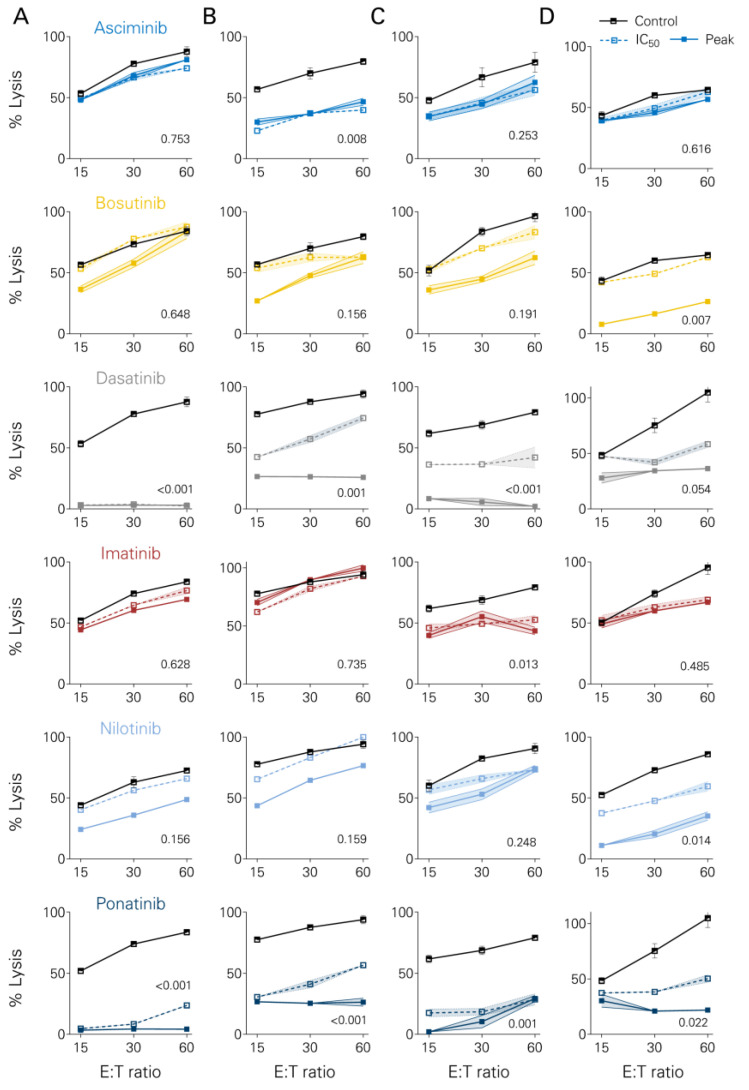
Short-term lysis of ALL cells by NK cells in the presence of TKI. Europium-loaded ALL cell lines were cultured for 4 h together with PBMCs (*n* = 3) and Rituximab +/− different TKI (Asciminib, Bosutinib, Dasatinib, Imatinib, Nilotinib, Ponatinib) at effector:target (E:T) ratios of 15, 30 and 60. Europium release was measured as a marker for tumor cell lysis. Solid lines indicate TKI at peak values and dotted lines indicate conditions with IC50 levels. The following cell lines were used: (**A**) BCR-ABL1-negative Nalm-6. (**B**) BCR-ABL1-negative Nalm-16. (**C**) BCR-ABL1-positive SD-1. (**D**) BCR-ABL1-positive TOM-1. Boxplots with Tukey whiskers. ANOVA.

Short-term lysis of B-ALL cells by NK cells was assessed using europium kill assays (Figure 4). ASCI moderately inhibited tumor cell lysis in one out of four cell lines (Nalm-16, *p* = 0.008). Similarly, BOSU decreased tumor cell killing at peak levels in one cell line (TOM-1, *p* = 0.007). DASA dose-dependently inhibited tumor cell killing in three out of four cell lines (Nalm-6, *p* < 0.001; Nalm-16 *p* < 0.001; SD-1 *p* < 0.001). A moderate decrease in tumor cell lysis was observed with IMA only in SD-1 (*p* = 0.013). NILO impaired killing in TOM-1 (*p* = 0.014), whereas PONA consistently blocked tumor cell lysis in all cell lines (*p* = 0.022 in TOM-1, *p* < 0.001 in other cell lines). 

### 3.5. Long-Term Lysis in the Presence of TKI

**Figure 5:**
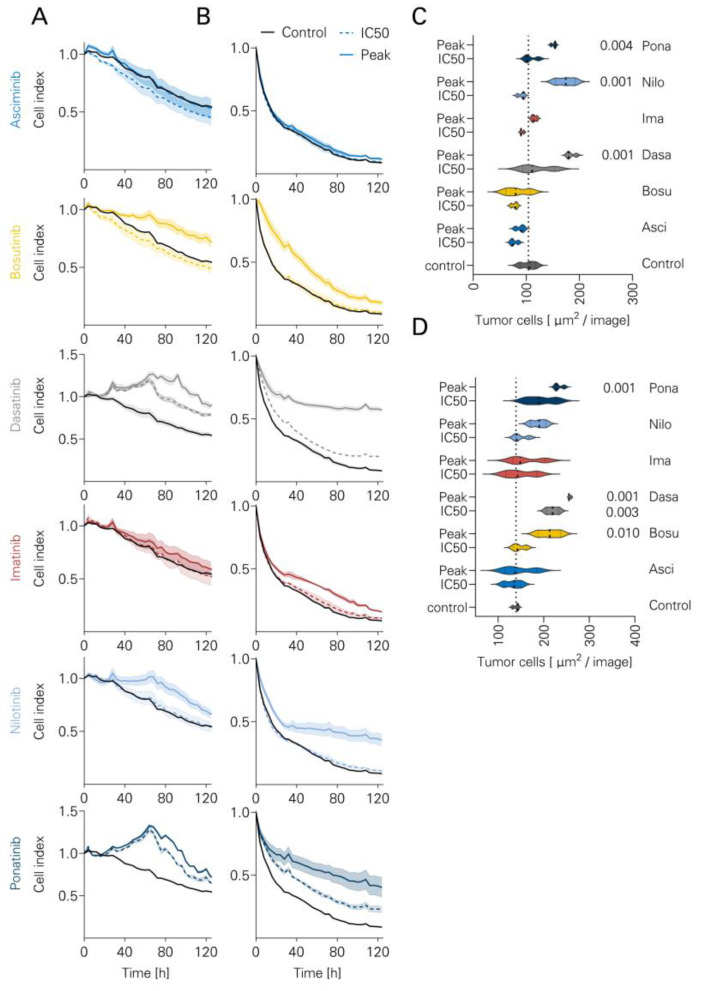
Long-term lysis of ALL cells by NK cells in the presence of TKI. ALL cell lines were cultured in an Incucyte real-time assay system for 120 h together with PBMCs (*n* = 3) and Rituximab +/− different TKI (Asciminib, Bosutinib, Dasatinib, Imatinib, Nilotinib, Ponatinib). Cell indices indicate the presence of viable tumor cells. Solid lines indicate TKI at peak values and dotted lines indicate conditions with IC50 levels. The following cell lines were used: (**A**) BCR-ABL1-negative Nalm-6. (**B**) BCR-ABL1-positive SD-1. (**C**,**D**) Absolute numbers of viable tumor cells after 120 h coculture with NK cells, rituximab +/− TKI are shown (*n* = 3). Violin plots. The following cell lines are shown: (**C**) Nalm-6, (**D**) SD-1. ANOVA with Bonferroni correction.

To further investigate the long-term inhibitory effects of TKI, Incucyte live cell imaging assays were performed for 120 h using Nalm-6 (Figure 5A) and SD-1 (Figure 5B). Exemplary sample snapshots are shown in Supplementary Figure S2. Consistent with the short-term results, IMA and ASCI did not inhibit long-term lysis. NILO inhibited SD-1 killing at peak levels (*p* = 0.001). PONA inhibited lysis of both Nalm-6 and SD-1 at peak levels (*p* = 0.001 and 0.004, respectively). BOSU inhibited killing of Nalm-6 only at peak levels (*p* = 0.010). DASA inhibited killing of Nalm-6 at both concentration levels (IC50 *p* = 0.003, peak *p* = 0.001). In addition, DASA inhibited lysis of SD-1 at peak levels (*p* = 0.001). Results for Nalm-16 cells and TOM-1 are shown in Supplementary Figure S3.

Finally, we investigated whether NK cell stimulation with IL-15 could help to overcome the inhibitory effects of TKI. Interestingly, NK cell proliferation observed with IL-15 could be additionally boosted in the presence of DASA (Supplementary Figure S4). Similar effects were not observed with other TKI.

In summary, DASA and PONA exhibited the most profound inhibitory effects on NK cell activation, degranulation and tumor cell killing. The other TKI had little or no effect on rituximab-mediated ADCC.

## 4. Discussion

Patients carrying the BCR-ABL1 fusion gene represent a high-risk population within the B-ALL patient cohort. Recently, chemotherapy-sparing regimens combining TKI and immunotherapeutic agents have shown promising efficacy in these patients. However, the interplay between immunotherapy and TKI therapy remains incompletely understood. We report on the interference of the classical ATP site TKI and the STAMP inhibitor ASCI with rituximab-induced ADCC.

Asciminib is currently approved in Europe for the treatment of patients with chronic myeloid leukemia who have been treated with at least two different TKIs. Its mechanism of action is based on binding to an allosteric site, in contrast to the ATP site TKIs. Common side effects include bone marrow toxicity, elevated liver enzymes, diarrhea, rash, QTc prolongation and nausea [28]. Its use may be limited in certain patients due to its weak inhibition of CYP3A and CYP2C9 [29]. Emerging evidence suggests clinical activity of ASCI in B-ALL. In one case report, ASCI was successfully used in combination with PONA in a patient with relapsed BCR-ABL1-positive B-ALL [9]. A phase 1 study in 14 patients evaluated ASCI in combination with dasatinib and prednisone for the same indication [19]. Molecular responses were observed in 78.6% of patients after three cycles. In vitro data show that the efficacy of ASCI in NUP214-ABL1 mutated cases appears to depend on the presence of an intact ABL1 SH3 domain [30]. In vitro data from refractory cases suggest that combination treatment of ASCI with an ATP site TKI can restore efficacy in ATP site mutated cases resistant to PONA [21].

Monoclonal antibody therapy with rituximab is an important component of B-ALL therapy. CD20 expression itself is a negative prognostic marker in adult B-ALL [31] and CD20 positivity is generally defined as CD20 expression in >20% of blasts. A recent study suggests increased CD20 expression in BCR-ABL1 positive cases [5]. The addition of 16–18 doses of rituximab to multi-agent chemotherapy in a clinical trial by the GRAALL group comprising 209 patients with CD20 positive B-ALL resulted in significantly prolonged event-free survival [12]. Rituximab plus Hyper-CVAD (fractionated cyclophosphamide, vincristine, doxorubicin, dexamethasone) resulted in a complete remission rate of 95% [13]. More recent data suggest that baseline CD20 expression may not be an optimal selection factor for the use of rituximab, as CD20 is upregulated upon application of steroids [32]. The same clinical trial of 586 patients applied four doses of rituximab regardless of CD20 expression and found no survival benefit. The authors suggest that a higher number of doses should be applied [32]. The Fc-optimized anti-CD19 antibody tafasitamab was tested in a phase 2a clinical trial in 22 B-ALL patients. The overall response rate was 9%, with one CR and one MRD-negative Cri [14]. These data suggest clinical activity, but also the lack of efficacy as a single-agent therapy. Therefore, combinatorial regimen should be evaluated. 

Data on ASCI in combination with NK cell-based immunotherapy are scarce. Domka et al. showed similar results in B-ALL cell lines and in xenografts with NSG mice and primary patient B-ALL cells [5]. Consistent with our data, DASA and PONA inhibited NK cell function, whereas ASCI did not. BOSU, DASA, IMA, NILO and PONA target the ATP site of BCR-ABL and affect multiple off-target kinases with varying affinity. BOSU affects the phosphorylation of SRC, ERK, S6 and STAT3 [33]. DASA inhibits the phosphorylation of ERK and PI3K [17]. IMA has been reported to inhibit c-kit and PDGFR signaling [34,35]. NILO has been shown to inhibit NK cell function in part through direct toxicity [17]. PONA has been shown to inhibit PDGFRα, VEGFR2, FGFR1 and SRC [36,37]. For example, studies have shown that blocking SRC, ERK or PI3K can reduce the cytotoxic activity of natural killer cells [38,39,40]. In contrast, ASCI is reported to be a highly selective allosteric inhibitor of BCR-ABL by blocking the myristoyl pocket. It has not shown any known off-target effects on other kinases at relevant concentrations [41,42]. Variations in the effects of the tested TKIs on the cytotoxic function of NK cells in the short- and long-term may be due to differences in their targeting of off-target kinases, which may alter multiple signaling pathways.

There are some similarities between the TKI-related inhibition of bispecific antibody-mediated T cell activation by blinatumomab and inhibition of ADCC. T cell proliferation and activation is largely affected by DASA and PONA and to a lesser extent by NILO [43,44]. However, the in vitro data showing T cell inhibition stand in contrast to the high efficacy of the regimen consisting of DASA/PONA and the bispecific CD19xCD3 antibody blinatumomab [10,45]. The differences between in vitro data and clinical efficacy may be due to the short half-life of TKI and the resulting differences between in vitro dosing and in vivo pharmacokinetics [43]. As this manuscript reports in vitro data, NK cell inhibition may not be seen in patients to the same extent, which is a limitation of our data. Recent in vitro data suggest that T cell activation and metabolic fitness are not affected by ASCI [46]. In contrast to the in vitro data on T cells, the NK cell activation we observed seems to be more robust even in the presence of an inhibitory TKI. Furthermore, certain effector molecules were not affected by TKI, whereas T cell-derived cytokines are strongly affected by co-treatment with TKI [43,44]. While blinatumomab has revolutionized the treatment of B-ALL, the timing of bispecific antibody therapy and chemotherapy remains unclear. In contrast, monospecific antibodies are easily combined with a variety of chemotherapeutic agents, but lack efficacy as monotherapies [14].

Given the results presented here and their relevance, there is ample room for further research. An evaluation of the relevant effects of the TKIs described here in an in vivo model would strengthen the conclusions drawn. This would also help to clarify the effects of other mechanisms of action induced by rituximab. For example, antigen-dependent cellular phagocytosis by macrophages, complement-dependent cytotoxicity and effector functions of neutrophils are not reflected in our setting, but may help to further validate and strengthen our findings.

The interplay between TKI and immunotherapy is complex. We report that IL-15 can overcome the inhibitory effects of DASA on NK cells and that the combination may induce further NK cell proliferation. Monoclonal antibody therapy may therefore be combined with additional cytokine treatments. Further animal studies and clinical trials incorporating pharmacokinetics are needed to fully evaluate the potential of combining TKI and monoclonal antibody therapy in BCR-ABL1-positive B-ALL patients. Given the non-interference of ASCI with ADCC, a head-to-head trial evaluating different TKI in BCR-ABL1-positive B-ALL should be performed.

## 5. Conclusions

To the best of our knowledge, we here provide the first full-length report on the effects of ASCI on NK cell-mediated ADCC in B-ALL. Our data support further preclinical and clinical analysis of ASCI in B-ALL.

## Data Availability

The data presented in this study are available on request from the corresponding author.

## Supplementary Materials

The following supporting information can be downloaded at: https://www.mdpi.com/article/10.3390/cancers16071288/s1, Figure S1: Flow cytometric gating strategy for degranulation assays; Figure S2: Exemplary data from Incucyte long-term lysis assays; Figure S3: Long-term lysis of ALL cells by NK cells in the presence of TKI; Figure S4: NK cell proliferation induced by IL-15 in the presence of TKI.
