# Supplementary material for: Asciminib Maintains Antibody-Dependent Cellular Cytotoxicity against Leukemic Blasts
Source: Cancers (Basel). 2024 Mar 26;16(7):1288. doi: 10.3390/cancers16071288 (PMC11010908; doi:10.3390/cancers16071288)
Supplement: Supplementary file 1 [file cancers-16-01288-s001.zip › cancers-2901521-supplementary.pdf]

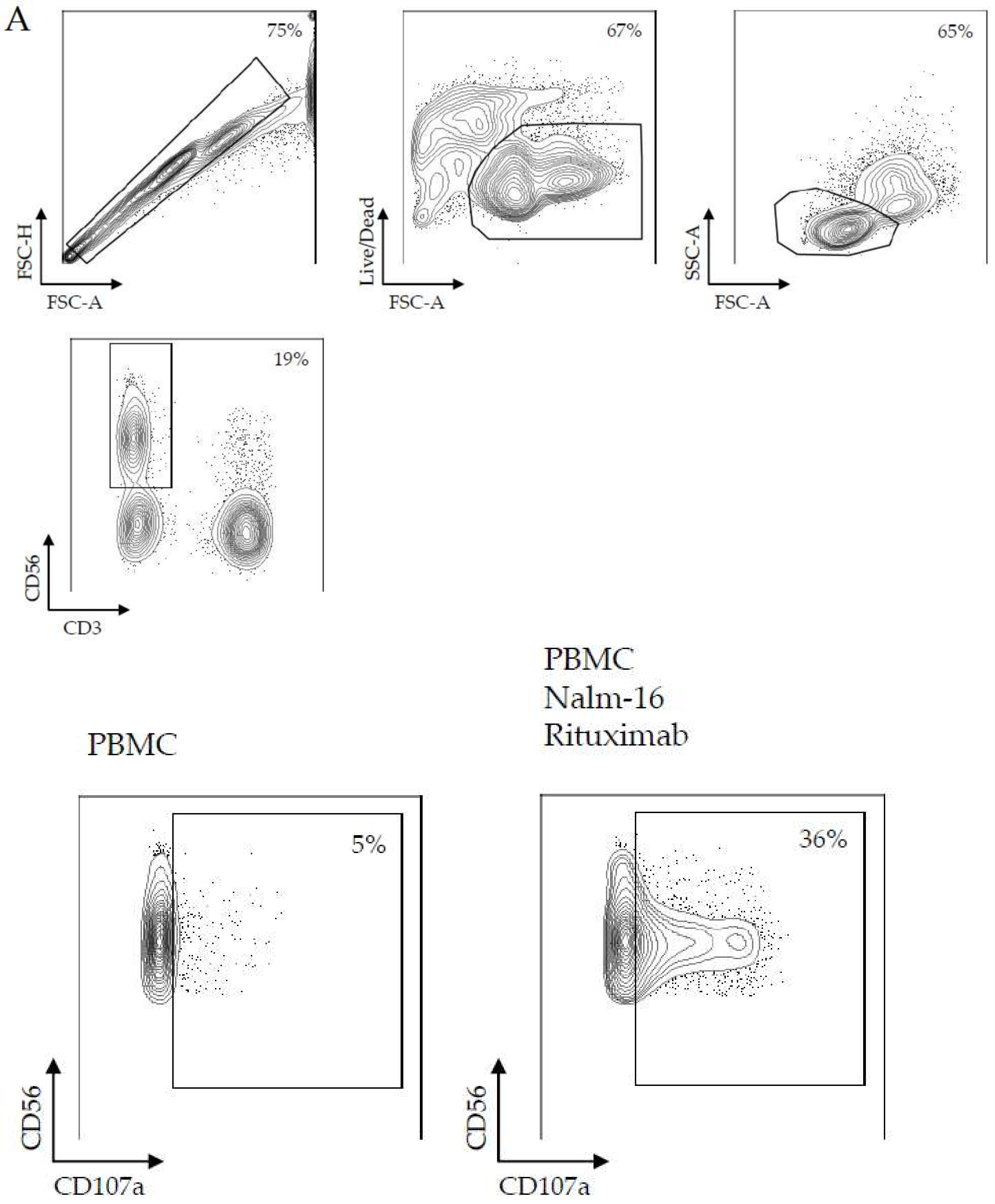

SUPPLEMENTARY FIGURE S1 Flow cytometric gating strategy for degranulation assays. Co-cultures of PBMC and tumor cells (exemplified by Nalm-16) were gated as follows: singlets, live cells, lymphocytes, CD56+CD3-NK cells, CD107a as surrogate marker for degranulation.

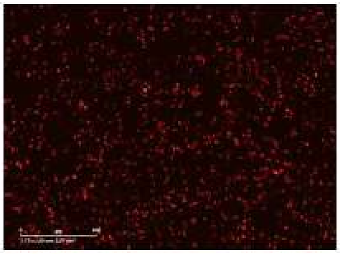

Nalm-16
PBMC

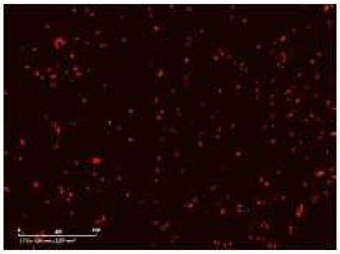

Nalm-16
PBMC
Rituximab

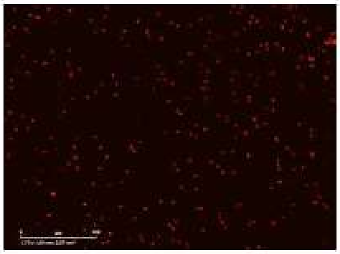

Nalm-16
PBMC
Rituximab
Asciminib Peak

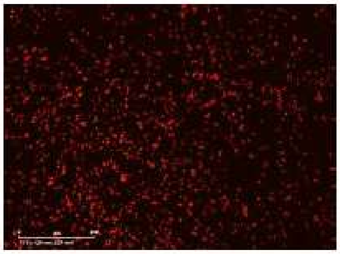

Nalm-16
PBMC
Rituximab
Bosutinib Peak

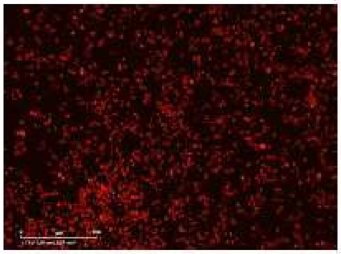

Nalm-16
PBMC
Rituximab
Dasatinib Peak

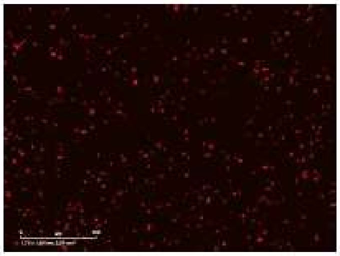

Nalm-16
PBMC
Rituximab
Imatinib Peak

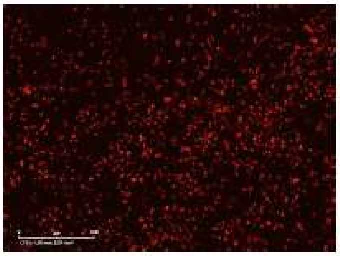

Nalm-16
PBMC
Rituximab
Nilotinib Peak

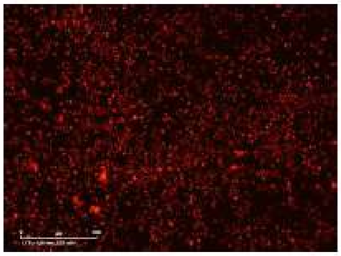

Nalm-16
PBMC
Rituximab
Ponatinib Peak

SUPPLEMENTARY FIGURE S2 Exemplary data from Incucyte long-term lysis assays. **A** Snapshots showing the abundance of viable tumor cells (red fluorescent) after 80 hours under different conditions.

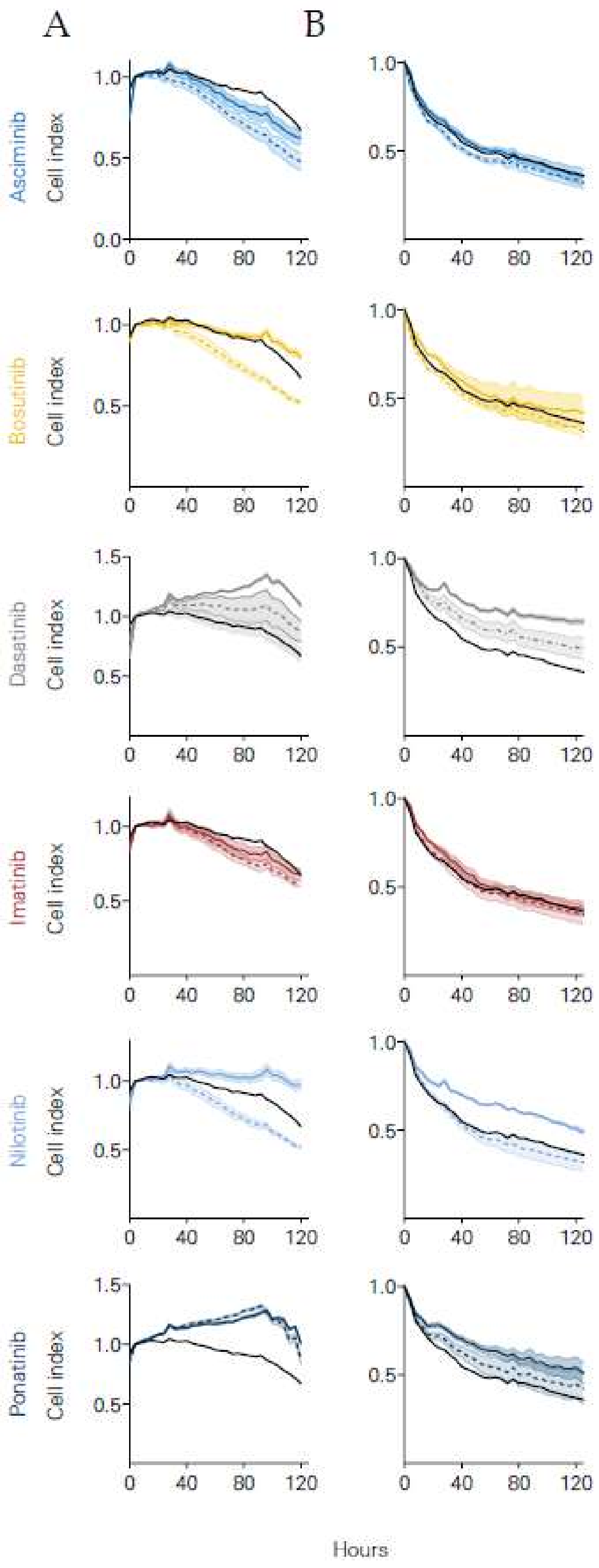
A
B
Asciminib
Bosutinib
Dasatinib
Imatinib
Nilotinib
Ponatinib
Cell index
Hours

C

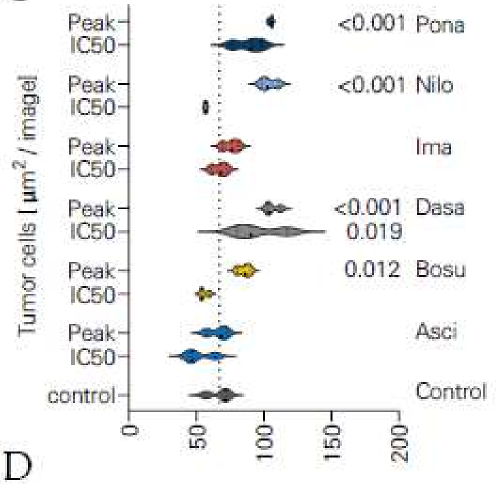

D

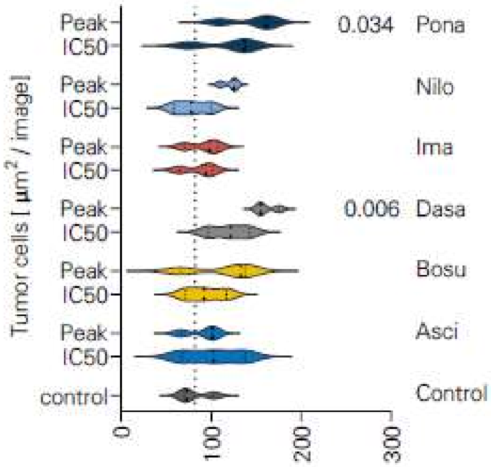

SUPPLEMENTARY FIGURE S3 Long-term lysis of ALL cells by NK cells in the presence of TKI. ALL cell lines were cultured in an Incucyte real-time assay system for 120 hours together with PBMC (n=3) and Rituximab +/- different TKI (Asciminib, Bosutinib, Dasatinib, Imatinib, Nilotiib, Ponatinib). Cell indices indicate the presence of viable tumor cells. The following cell lines were used: **A** BCR::ABL1-negative Nalm-16. **B** BCR::ABL1-positive TOM-1. **C-D** Absolute numbers of viable tumor cells after 120 h co-culture with NK cells, rituximab +/- TKI are shown. The following cell lines are shown: **C** Nalm-16, **D** TOM-1. ANOVA with Bonferroni correction.

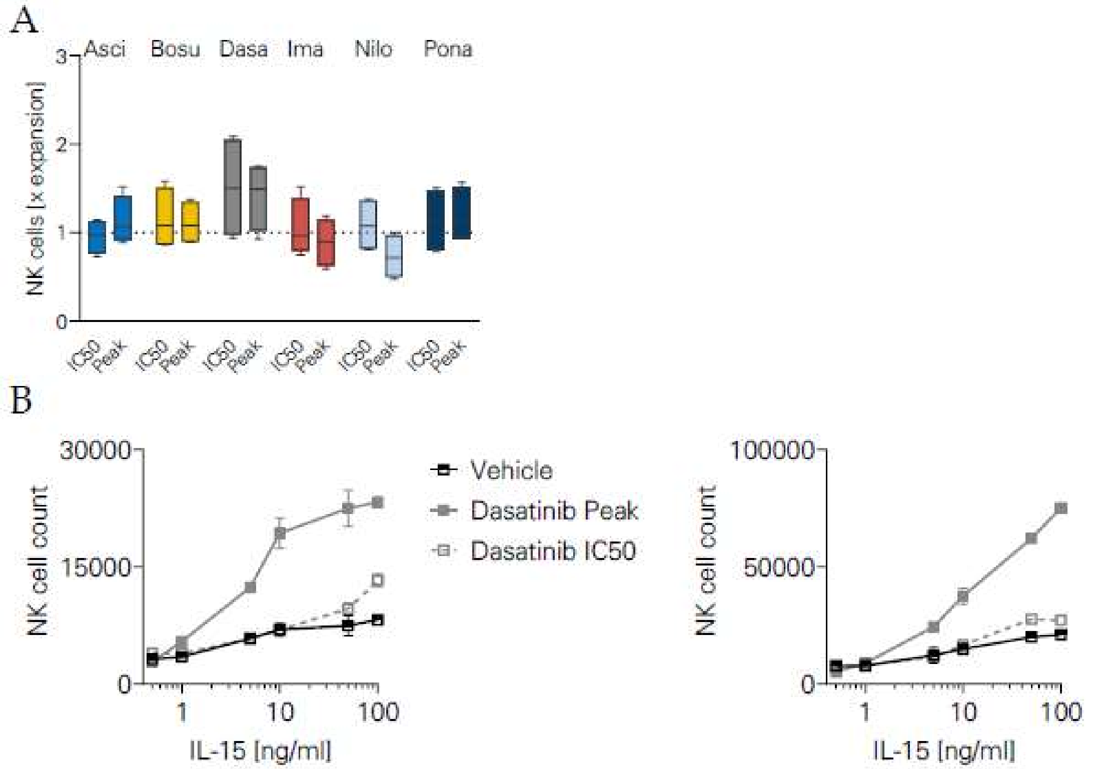

SUPPLEMENTARY FIGURE S4 NK cell proliferation induced by IL-15 in the presence of TKI. PBMC (n=4) were cultured for 7 days with IL-15 at 10ng/ml +/- different TKI (Asciminib, Bosutinib, Dasatinib, Imatinib, Nilotiib, Ponatinib). **A** Relative NK cell expansion with PBMC + IL-15 + TKI versus PBMC + IL-15 (control, set to 1.0). **B** Exemplary dose curves indicating NK cell proliferation in the presence of varying IL-15 doses +/- dasatinib.
